# Human Chondrocytes Respond Discordantly to the Protein Encoded by the Osteoarthritis Susceptibility Gene *GDF5*

**DOI:** 10.1371/journal.pone.0086590

**Published:** 2014-01-21

**Authors:** Madhushika Ratnayake, Frank Plöger, Mauro Santibanez-Koref, John Loughlin

**Affiliations:** 1 Musculoskeletal Research Group, Institute of Cellular Medicine, Newcastle University, Newcastle upon Tyne, United Kingdom; 2 Biopharm GmbH, Heidelberg, Germany; 3 Institute of Genetic Medicine, Newcastle University, Newcastle upon Tyne, United Kingdom; University of Northampton, United Kingdom

## Abstract

A genetic deficit mediated by SNP rs143383 that leads to reduced expression of *GDF5* is strongly associated with large-joint osteoarthritis. We speculated that this deficit could be attenuated by the application of exogenous GDF5 protein and as a first step we have assessed what effect such application has on primary osteoarthritis chondrocyte gene expression. Chondrocytes harvested from cartilage of osteoarthritic patients who had undergone joint replacement were cultured with wildtype recombinant mouse and human GDF5 protein. We also studied variants of GDF5, one that has a higher affinity for the receptor BMPR-IA and one that is insensitive to the GDF5 antagonist noggin. As a positive control, chondrocytes were treated with TGF-β1. Chondrocytes were cultured in monolayer and micromass and the expression of genes coding for catabolic and anabolic proteins of cartilage were measured by quantitative PCR. The expression of the GDF5 receptor genes and the presence of their protein products was confirmed and the ability of GDF5 signal to translocate to the nucleus was demonstrated by the activation of a luciferase reporter construct. The capacity of GDF5 to elicit an intracellular signal in chondrocytes was demonstrated by the phosphorylation of intracellular Smads. Chondrocytes cultured with TGF-β1 demonstrated a consistent down regulation of *MMP1*, *MMP13* and a consistent upregulation of *TIMP1* and *COL2A1* with both culture techniques. In contrast, chondrocytes cultured with wildtype GDF5, or its variants, did not show any consistent response, irrespective of the culture technique used. Our results show that osteoarthritis chondrocytes do not respond in a predictable manner to culture with exogenous GDF5. This may be a cause or a consequence of the osteoarthritis disease process and will need to be surmounted if treatment with exogenous GDF5 is to be advanced as a potential means to overcome the genetic deficit conferring osteoarthritis susceptibility at this gene.

## Introduction

Growth differentiation factor 5 (GDF5) is closely related to the bone morphogenetic proteins (BMPs) and is a member of the transforming growth factor- β (TGF-β) superfamily. GDF5 is involved in bone and cartilage development, maintenance and repair and is a marker for early joint formation [1].

GDF5 signalling requires both BMP receptor type II (BMPR-II) and BMP receptor type IB (BMPR-IB). BMPR-II is constitutively active and upon ligand binding, transphosphorylates BMPR-IB [2]. This leads to the phosphorylation of the downstream Smad1/5/8 signalling molecules. Although GDF5 shows binding affinity towards BMP receptor type IA (BMPR-IA), GDF5 has been shown to exhibit a 17-fold higher affinity towards BMPR-IB, compared to BMPR-IA [2]. The phosphorylated Smad 1/5/8 then complexes with Smad 1/4, and the complex translocates into the nucleus and regulates transcription of target genes [3]. These targets include *COL2A1* and *ACAN*, which encode type II collagen and aggrecan, the principal structural proteins of cartilage [4]. GDF5 signalling can be negatively regulated by soluble proteins including noggin, which binds to GDF5 extracellularly and blocks its interaction with the cell surface receptors [5], [6].

The importance of GDF5 in the development of synovial joints has been shown in mouse models and human diseases. The presence of a frame shift mutation in *Gdf5* in the brachypodism mouse results in the inability to form (homozygote) or to maintain (heterozygote) normal synovial joint function [7]. Human chondrodysplasias caused by mutations in *GDF5* include Hunter-Thompson syndrome, Grebe syndrome and Brachydactyly type C, which are associated with various skeletal abnormalities [8].

As the role of GDF5 in skeletogenesis is well established, *GDF5* was examined in a candidate gene analysis aiming to identify genes that harbour osteoarthritis (OA) susceptibility alleles in Japanese and Han Chinese populations [9]. OA is characterised by the gradual focal loss of articular cartilage in synovial joints such as the hips and the knees, leading to full thickness lesions that expose the underlying subchondral bone. The authors identified a single nucleotide polymorphism (SNP) in the 5′ untranslated region (UTR) of *GDF5* (rs143383, C/T) as being associated with OA. The Asian report of OA association to rs143383 was quickly replicated in a European study [10]. Cartilage is synthesised and maintained by a single cell type, the chondrocyte, and reduced transcription in the chondrocytes of OA patients of the T-allele of rs143383 relative to its C- allele has been demonstrated [11]. Overall, these results suggest that the *GDF5* OA susceptibility is mediated through a reduction in expression of the gene.

Several animal studies have reported on the use of GDF5 in therapeutics. Studies in rats have shown that GDF5 can be used to stimulate tendon healing [12], [13]. GDF5 has also been shown to be effective in repair or slowing down the degeneration of intervertebral discs in mouse, rabbit and bovine models [14]–[17]. Also, it has been reported that GDF5 enhanced chondrogenic differentiation and hypertrophy of human MSCs, thus showing potential to be used as a therapeutic in fracture repair [18].

Two publications have shown promising results of the stimulatory effects of GDF5 on matrix synthesis in human articular chondrocytes *in vitro*. Bobacz *et al*. [19] showed an increase in glycosaminoglycan (GAG) synthesis in normal and OA chondrocytes cultured with GDF5, and an increase in *ACAN* mRNA levels. Chubinskaya *et al*. [20] observed an increase in GAG synthesis in alginate bead cultures of chondrocytes in the presence of GDF5.

The fact that the OA associated T-allele of rs143383 mediates reduced expression of *GDF5* has led to us hypothesising that one means of alleviating this genetic deficit could be via the supply of exogenous GDF5 to chondrocytes. Investigating the logistics of this is the aim of this report. Chondrocytes harvested from OA patients that had undergone total hip or total knee replacement surgery were cultured with or without recombinant GDF5. We then assessed whether this triggers expression changes of genes involved in metabolic processes in chondrocytes.

## Materials and Methods

### Ethics Statement and Cartilage Samples from OA Patients

The Newcastle and North Tyneside research ethics committees granted ethical approval for the collection of cartilage from patients undergoing hip or knee joint replacement for primary OA (REC reference number 09/H0906/72). Each donor provided informed consent. The project was discussed with the donor verbally by a trained research nurse and if the donor agreed to participate written consent was then taken. This consent procedure was approved by the ethics committee and the written consent was then filed by the consenting nurse. OA status was confirmed using pre-operative records. All patients had full-thickness cartilage lesions.

### Cartilage Digestion and Chondrocyte Culture with Exogenous GDF5

Chondrocytes were isolated by enzymatic digestion of cartilage as previously described [21]. The cells were cultured in DMEM culture media supplemented with 10% FBS, 2 mM glutamine, 100 U/ml penicillin, 100 µg/ml streptomycin and 50 U/ml nyastatin.

For western blot analysis, chondrocytes were cultured at 350,000 cells/well in 6-well cell culture plates in 2 ml of culture media. Once 80% confluent, the cells were cultured in serum-free media overnight, before stimulation with 100 ng/ml of each of the GDF5 proteins. Cells were isolated at four different time points after stimulation (15 minutes, 30 minutes, 1 hour and 2 hours). TGF-β1 (5 ng/ml) stimulation for one hour was used as a positive control.

For gene expression analysis, chondrocytes were cultured in monolayer and as high-density micromass. For monolayer culture, chondrocytes were cultured at 10,000 cells/well in 96-well cell culture plates in 200 µl of DMEM culture media until 80% confluent. Cells were then cultured in serum-free media overnight. Five plates were established since the cells were to be isolated at five different time points (0 hours, 6 hours, 12 hours, 24 hours and 48 hours). Six wells per time point were used for each treatment group (untreated, mouse GDF5, human wild type GDF5 and the two variants A and B). For micromass culture, chondrocytes were cultured in wells of a 24-well culture plate at a density of 400,000 cells/well in 20 µl droplets of media. Four replicates were prepared for each treatment group (untreated, 100 ng/ml mouse, human wildtype GDF5, human GDF5 variants A and B, and 5 ng/ml human TGF-β1). The cells were incubated at 37°C for 2 hours and 400 µl of media containing the growth factors was then added and the cells were incubated for 5 days. In all experiments, untreated control wells received serum-free media and cells were incubated at 37°C until the time points were reached. Details regarding the 25 patients studied using monolayer analysis and the 19 patients studied using micromass analysis are listed in supplementary Tables S1 and S2 respectively.

### Gene Expression Analysis Using Quantitative Real Time PCR

TaqMan primers and probes were used to analyse gene expression changes in a panel of genes (Table S3). Gene expression was measured relative to the housekeeping genes *GAPDH*, *HPRT1* and *18S*. Reactions were performed on an ABI PRISM 7900HT Real Time PCR System. The relative expression for each gene was analysed using the comparative cycle threshold (Ct) method using SDS 2.3 software (Applied Biosystems).

For gene expression analysis directly from cartilage tissues, 1 µg of RNA was extracted from the tissues as described previously [11]. cDNA was then synthesised using a SuperScript First-Strand cDNA Synthesis kit (Invitrogen) according to the manufacturer’s instructions.

For gene expression analysis of chondrocytes cultured in monolayer, at appropriate time points the cells were lysed using 30 µl of Cells-to-cDNA II Cell Lysis Buffer (Ambion) at 75°C for 15 minutes. Eight µl of the cell lysate was reverse transcribed using First-Strand cDNA Synthesis kit using M-MLV reverse transcriptase (Invitrogen) according to the manufacturer’s instructions.

For gene expression analysis of chondrocytes cultured in micromass, the cells were washed in PBS and scraped off the surface of the well using a pipette tip. The cells in PBS were centrifuged at 13,000 rpm for 5 minutes to obtain a cell pellet. RNAs were isolated from the cell pellets using the Trizol/chloroform method according to manufacturer’s guidelines (Invitrogen) and were taken forward for cDNA synthesis.

### Western Blot Analysis

Cells were lysed in 30 µl of protein lysis buffer (50 mM Tris, 10% v/v glycerol, 50 mM NaF, 1 mM EGTA, 1 mM EDTA, 10 mM glycerol phosphate, 1% v/v Triton X-100, 1**×** complete inhibitor cocktail, 1 µM microcystin-LR and 1 mM Na_3_VO_4_). Ten µg of protein were electrophoresed through a 12% bis-acrylamide gel and transferred to a polyvinylidene fluoride membrane (GE Healthcare). The membrane was then blocked and probed with antibodies against BMPR-II (goat polyclonal; Santacruz, cat no. sc-5682), BMPR-IA (rabbit polyclonal; Santacruz, cat no. sc-20736), BMPR-IB (rabbit polyclonal; Santacruz, cat no. sc-25455) or phospho-Smad1 (Ser463/465)/Smad5 (Ser463/465)/Smad8 (Ser426/428; rabbit polyclonal; Cell Signalling, cat no. 9511), overnight. Anti-β-actin (Sigma, cat no. A5316) antibody was used as a loading control. Enhanced chemiluminescent reagent (GE Healthcare) was used for visualisation of the proteins using a G:BOX gel doc system (Syngene).

### Transfection of SW1353 Cells with Smad 1/5/8 Luciferase Reporter Vector

Cells from the human chondrosarcoma cell line SW1353 [22] were cultured at 17, 500 cells/well in 48-well cell culture plates in 300 µl of DMEM/F-12 media (Gibco) containing 10% FBS to 80% confluence. Cells were co-transfected with 15 ng of *Renilla* vector DNA (Promega) as an internal control and 500 ng of basic pGL3-Smad responsive reporter vector DNA using ExGen 500 *in vitro* transfection reagent (Fermentas) following the manufacturer’s protocol. The Smad responsive reporter vector contains six consecutive copies of the Smad-binding element, 5′-CCCGTCTGCCCCAGCCCAGACACCGTCGACCAAC-3′ [23]. The cells were incubated at 37°C for 24 hours.

### Stimulation of Cells with Exogenous Growth Factors (GDF5, TGF-β1 and BMP-2) Post Transfection

Twenty-four hours post transfection, the cells were incubated in 150 µl of serum-free media at 37°C overnight. The cells were stimulated with four different recombinant GDF5 proteins (mouse GDF5, R&D Systems; human wild type GDF5 and two variants A and B, Biopharm GmbH) at 10 ng/ml, 30 ng/ml, 100 ng/ml and 300 ng/ml. Recombinant human TGF-β1 (5 ng/ml; R&D Systems) and BMP2 (100 ng/ml; R&D Systems) were also used as positive controls. The experiment was performed in four replicates. The plates were incubated at 37°C and lysed at 6 hours, 12 hours, 24 hours and 48 hours post stimulation. All of the proteins are the active forms of the signalling molecules, free of the latency-associated peptides.

### Luciferase Reporter Assay

The cells were lysed at each time point using 65 µl of 1**×** Passive Lysis Buffer (Promega). Dual-Luciferase Reporter assay system (Promega) was used to measure luciferase activity in the transfected cells, following manufacturer’s instructions.

### Statistical Analysis

GraphPad Prism software was used for generating graphs and performing statistical analyses. A P-value of less than 0.05 was considered significant.

For luciferase activity measurements, the absorbance for firefly luciferase was divided by that for *Renilla* luciferase. A two tailed student’s *t-*test was used to compare the normalised values for untreated cells versus cells stimulated with exogenous growth factors.

For gene expression analyses, the gene expression relative to housekeeping genes (2∧-(Ct of target gene – average Ct of housekeeping genes)) was calculated for each treatment. A two tailed student’s *t-*test was performed to compare the untreated cells against the treated cells. The fold change in gene expression between the treated and untreated cells was calculated for each patient. A Wilcoxon signed rank test was then performed on the fold change in gene expression for each gene analysed, to determine if the data deviate significantly from a hypothetical value of 1, which signifies no change in gene expression.

## Results

### GDF5 Receptor Genes are Expressed in OA Cartilage and in Cultured OA Chondrocytes

**Figure 1:**
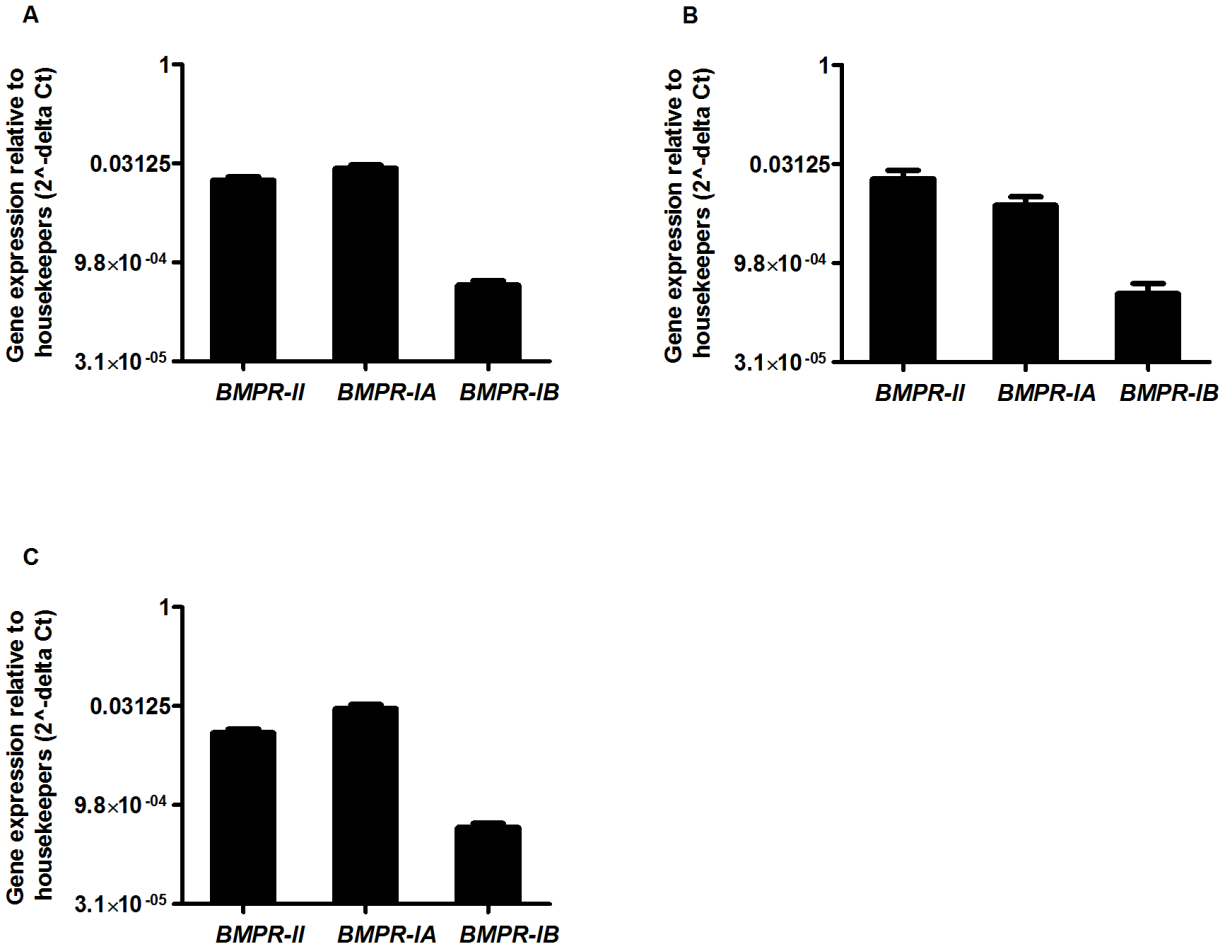
GDF5 receptor gene expression. Gene expression was measured in cartilage (A), in monolayer culture (B) and in micromass culture (C) relative to the housekeepers, *18S*, *GAPDH* and *HPRT1*. The error bars represent the standard error of the mean.

In order to investigate the effect of adding exogenous GDF5 to chondrocytes, it was important to first assess whether chondrocytes from OA patients expressed the genes *BMPR-II*, *BMPR-IA* and *BMPR-IB*, which encode the receptors that GDF5 binds to. We therefore measured the expression of these receptor genes by quantitative real time PCR (qPCR) using cDNA synthesised from RNA extracted directly from the cartilage tissue of 10 OA patients (3 hip and 7 knee). All three receptor genes, in particular *BMPR-II* and *BMPR-IA,* were expressed (Figure 1A). The expression of *BMPR-IA*, which encodes the alternative type I receptor that GDF5 binds to, was 60 fold (P = 7.2**×**10^−7^) higher compared to *BMPR-IB*, which encodes the receptor towards which GDF5 shows preferential activity. There was no significant difference (P<0.05) in the expression of *BMPR-II*, *BMPR-IA* or *BMPR-IB* between hip and knee cartilage.

We next measured the gene expression of *BMPR*-*II*, *BMPR-IA* and *BMPR-IB* in chondrocytes that had been extracted from the cartilage of 5 OA patients and then cultured in monolayer (Figure 1B), and in chondrocytes that had been extracted from the cartilage of 8 OA patients and then cultured in micromass (Figure 1C). In both instances the cells showed a similar gene expression pattern to that observed in cartilage tissue, with a 22 fold (P = 0.02) higher *BMPR-IA* expression compared to *BMPR-IB* expression in monolayer and a 65 fold (P = 1.3**×**10^−5^) higher *BMPR-IA* expression compared to *BMPR-IB* expression in micromass.

Protein extracted from chondrocytes from the cartilage of 3 OA patients were analysed by western blot to determine if BMPR-II, BMPR-IA and BMPR-IB proteins were expressed in these cells. The receptor expression pattern correlated with gene expression analyses, such that the cells showed higher levels of expression of BMPR-II and BMPR-IA compared to BMPR-IB (Figure S1).

### Four GDF5 Proteins were Examined

The above results demonstrated that the expression of *BMPR-IA* and its protein product was higher in chondrocytes compared to expression of *BMPR-IB* and its protein product. Therefore, we chose to include in our study a recombinant human variant form of GDF5 that was designed by Biopharm GmbH (Heidelberg, Germany) to show increased specificity for BMPR-IA compared to BMPR-IB (variant A). In addition, we used a second variant form of GDF5, also designed by Biopharm that is insensitive to the GDF5 antagonist noggin (variant B). Therefore, we used four different recombinant GDF5 proteins in total: wildtype mouse (R&D Systems), wildtype human (Biopharm) and the two variants, A and B. All four are the mature form of GDF5. The amino acid sequence difference between the mouse and human GDF5 is a single amino acid: asparagine at position 380 in mouse is a threonine at the comparable position (386) in human. Table 1 shows the sequence of the four GDF5 proteins encompassing the changes introduced in variants A and B. Variant A was designed by introducing two point mutations in GDF5 to swap two methionine residues to valine residues at positions 453 and 456 [24], [25]. Variant B was designed by exchanging the asparagine residue at position 445 in GDF5 with threonine [26].

**Table 1:** The amino acid substitutions of GDF5 variants A and B.

**Wildtype** **mGDF5**	430 PLRSHLEPTNHAVIQTLMNSMDPESTPPTCCVPTRL 466
**Wildtype** **hGDF5**	436 PLRSHLEPTNHAVIQTLMNSMDPESTPPTCCVPTRL 472
**hGDF5** **variant A**	436 PLRSHLEPTNHAVIQTLVNSVDPESTPPTCCVPTRL 472
**hGDF5** **variant B**	436 PLRSHLEPTTHAVIQTLMNSMDPESTPPTCCVPTRL 472

The amino acid sequences from 430–466 for mouse GDF5 and from 436–472 for human GDF5 proteins are shown with the change in amino acid sequences in variant A and B highlighted in red. mGDF5, mouse GDF5; hGDF5, human GDF5.

### All Four GDF5 Proteins Elicit Smad Signalling at 100 ng/ml

It has been shown previously that the optimal dose of GDF5 to be used for the stimulation of chondrocytes is 100 ng/ml [27]. In order to test this, we carried out a dose response analysis using a Smad responsive reporter assay, which is under the transcriptional control of Smad binding elements that drive firefly luciferase transcription [23]. BMP2 (at 100 ng/ml) [20] and TGF-β1 (at 5 ng/ml) [28] treatments were used as positive controls. We initially performed these experiments in OA chondrocytes, but were unsuccessful at transfecting these cells with the reporter. We therefore used instead the human SW1353 chondrosarcoma cell line [22] to carry out the dose response analyses. These cells show a similar GDF5 receptor gene expression profile to that observed in cartilage and in cultured chondrocytes, with a 15 fold (P = 0.0005) higher expression of *BMPR-IA* compared to *BMPR-IB* (Figure S2).

**Figure 2:**
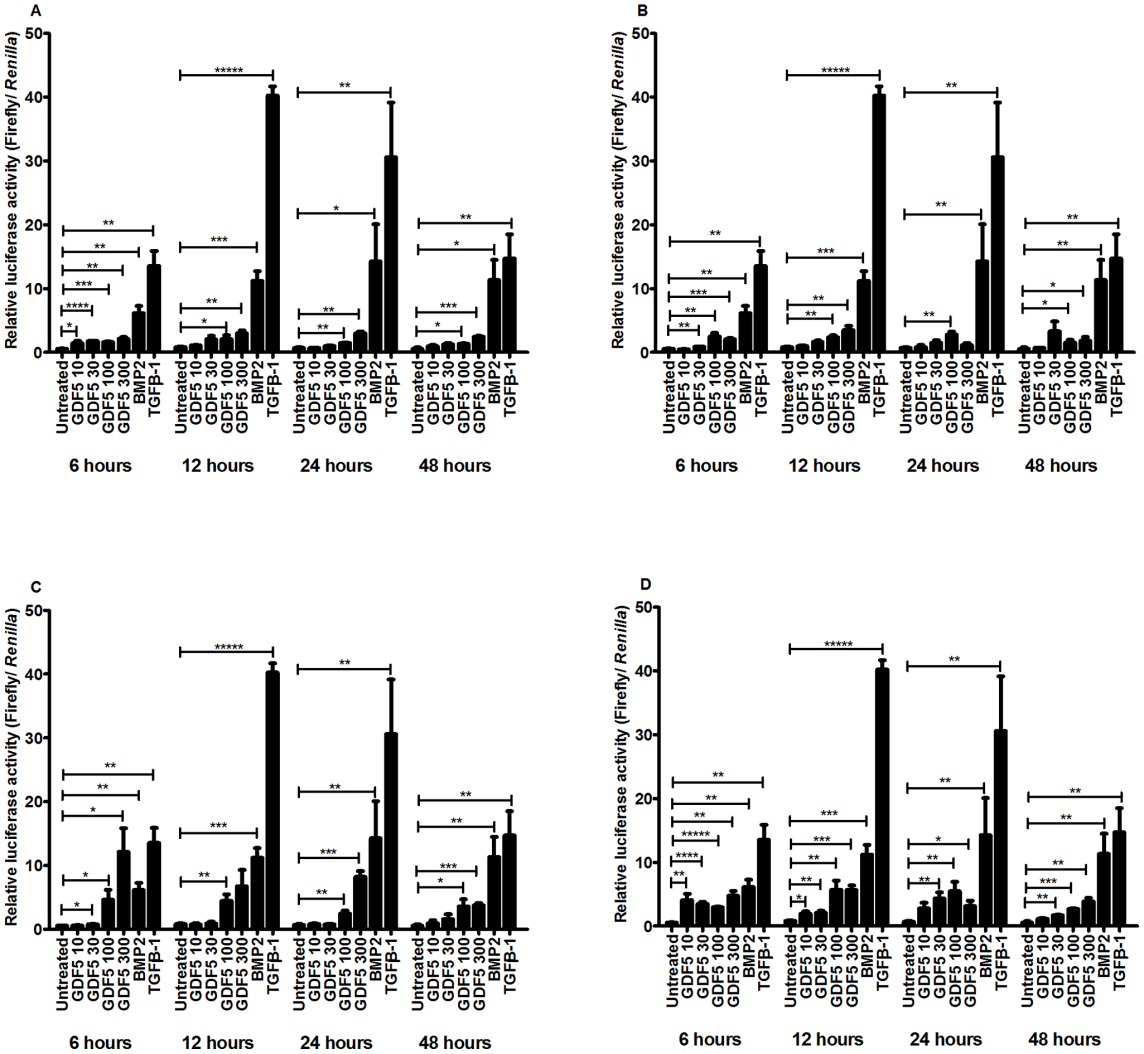
Dose response analysis using a Smad responsive reporter assay. Y-axis represents the luciferase activity readings generated in SW1353 cells in response to exogenous growth factor. Cells were stimulated for 6, 12, 24 and 48 hours with wildtype mouse GDF5 (A), wildtype human GDF5 (B), human GDF5 variant A (C) and human GDF5 variant B (D). BMP2 and TGF-β1 stimulations were used as positive controls. Error bars represent the standard error of the mean. GDF5 10, 10 ng/ml; GDF 30, 30 ng/ml; GDF5 100, 100 ng/ml; GDF5 300, 300 ng/ml. *P<0.05, **P<0.01, ***P<0.001, ****P<0.0001, *****P<0.00001, two-tailed Student’s t-test.

The cells stimulated with 10 ng/ml and 30 ng/ml of mouse GDF5 showed a significant increase in luciferase activity at 6 hours after stimulation, compared to untreated cells. However a significant effect was not seen at these doses at the two other time points analysed. The cells treated with 100 ng/ml and 300 ng/ml of mouse GDF5 showed a significant increase in luciferase activity compared to untreated cells at all time points analysed (Figure 2A).

Stimulation of cells with 10 ng/ml of wildtype human GDF5 did not increase luciferase activity compared to untreated cells at any of the time points analysed. Stimulation with 30 ng/ml increased luciferase activity only at 6 hours after stimulation. Cells cultured with 100 ng/ml GDF5 showed a significant increase in luciferase readings at all time points. Treatment with 300 ng/ml of wildtype human GDF5 resulted in a significant increase in luciferase activity at 6, 12 and 48 hour time points, but not at 24 hours (Figure 2B).

Stimulation of cells with 10 ng/ml of human GDF5 variant A did not result in a significant increase in luciferase activity, and cells stimulated with 30 ng/ml of variant A showed a significant increase only at 6 hours post stimulation. Cells cultured with 100 ng/ml of variant A showed a significant increase in luciferase readings at all time points. Stimulation with 300 ng/ml of variant A resulted in a significant increase in luciferase at 6, 24 and 48 hours post stimulation, but not at 12 hours (Figure 2C).

The cells cultured with 10 ng/ml of GDF5 variant B showed an increase in luciferase activity at 6 and 12 hours, but not at 24 or 48 hours after stimulation. The cells cultured with 30 ng/ml, 100 ng/ml and 300 ng/ml of this variant showed significantly higher luciferase activity at all time points (Figure 2D).

At all four time points, stimulation with BMP2 and TGF-β1 resulted in a significant increase in luciferase activity. Furthermore, BMP2 and TGF-β1 stimulations clearly elicited a greater response at all time points than any of the GDF5 proteins. Stimulation with 100 ng/ml of each of the four recombinant GDF5 proteins consistently and significantly increased luciferase activity at all time points. Furthermore, there was no significant difference between the luciferase readings generated in cells stimulated with 100 ng/ml and 300 ng/ml of GDF5 protein. As noted above, it has been shown that 100 ng/ml is the optimal concentration of recombinant GDF5 to be used in chondrocyte culture [27]. Based on our results and this previous data, we therefore chose to use 100 ng/ml GDF5 for the stimulation of OA chondrocytes in subsequent experiments.

### All Four GDF5 Proteins Activate Smad Signalling in OA Chondrocytes

**Figure 3:**
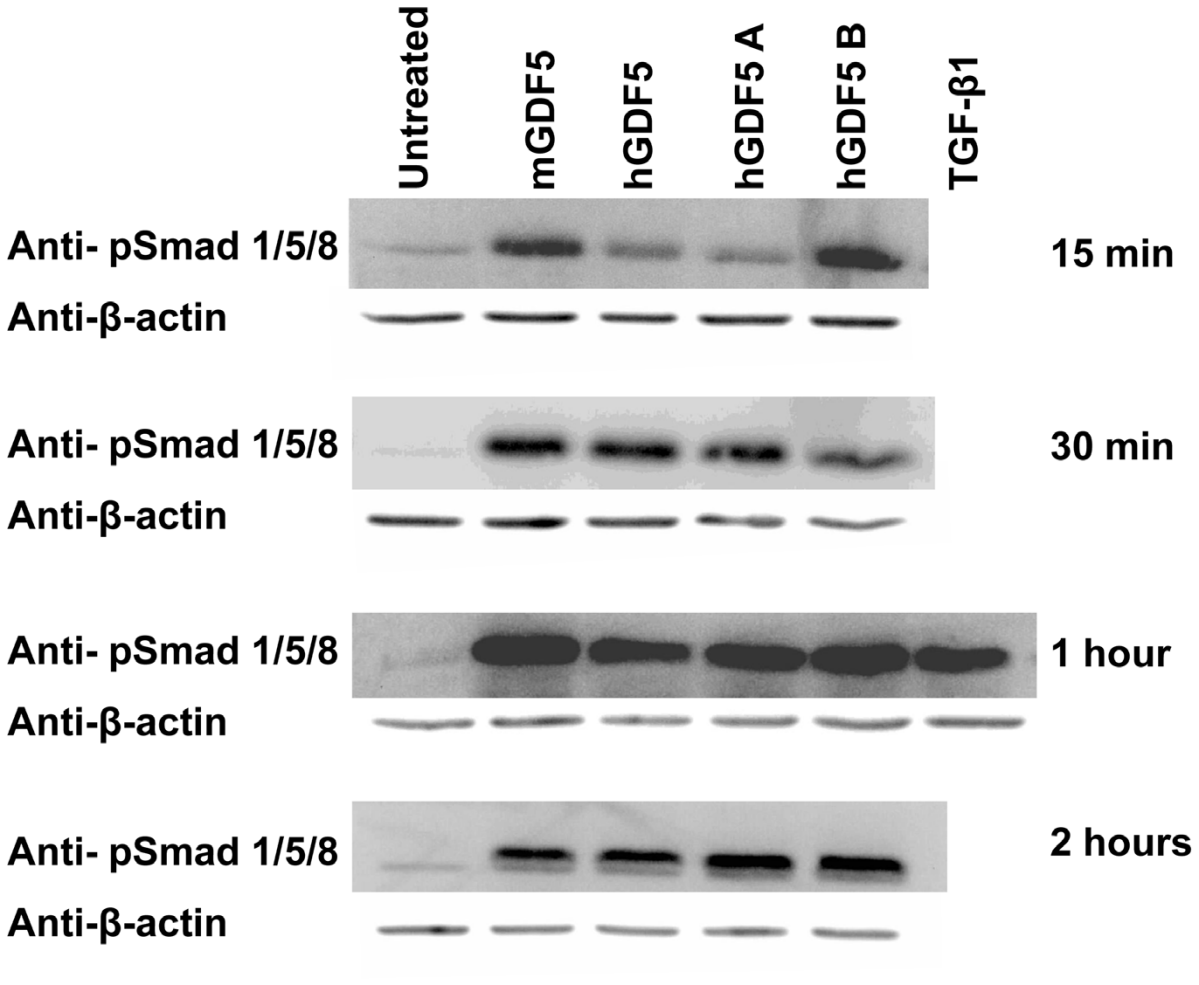
Activation of Smad signalling in OA chondrocytes after growth factor stimulation. Chondrocytes were cultured in monolayer and stimulated with each of the four GDF5 proteins for 15 minutes, 30 minutes, 1-β1 stimulation for 1 hour was used as a positive control. Protein was extracted and subjected to western blot analysis using an antibody against Smad 1/5/8 (anti-Smad1/5/8), with anti-β-actin antibody used as a loading control. This data comes from one OA patient. Identical data was obtained for a second OA patient (data not shown).

Protein was extracted from chondrocytes that had been grown in monolayer and stimulated with 100 ng/ml of each of the four GDF5 proteins for between 15 minutes and two hours. The protein was then subjected to western blot analysis using an antibody against phosphorylated Smad 1/5/8, with anti-β-actin antibody used as a loading control. The cells were also stimulated with TGF-β1 for 1 hour as a positive control. The Smad 1/5/8 signalling pathway was clearly activated in response to each GDF5 protein at all time points analysed (Figure 3).

### None of the GDF5s Elicit a Consistent Target Gene Response by OA Chondrocytes in Monolayer

Chondrocytes harvested from 25 OA patients (Table S1) were cultured in monolayer with (treated) or without (untreated) 100 ng/ml of the GDF5 proteins. Twleve of the 25 patients were cultured with wildtype mouse GDF5 (patients 1–9 and 21–23), three of whom were also separately cultured with human GDF5 and its two variants and with 5 ng/ml TGF-β1 (patients 21–23) and three separately with TGF-β1 (patients 7–9). Twelve of the 25 were cultured with wildtype human GDF5 (patients 10, 11,14–18 and 21–25), five of whom were also separately cultured with variant A and separately with variant B (patients 14–18) and two with human GDF5 variants and TGF-β1 (patients 24–25). Two of the 25 were cultured with variant A and separately with variant B (patients 12 and 13). Finally, two of the 25 were cultured only with TGF-β1 (patients 19 and 20). None of the culturing was performed with two or more of the growth factor proteins in the same culture mix.

**Figure 4:**
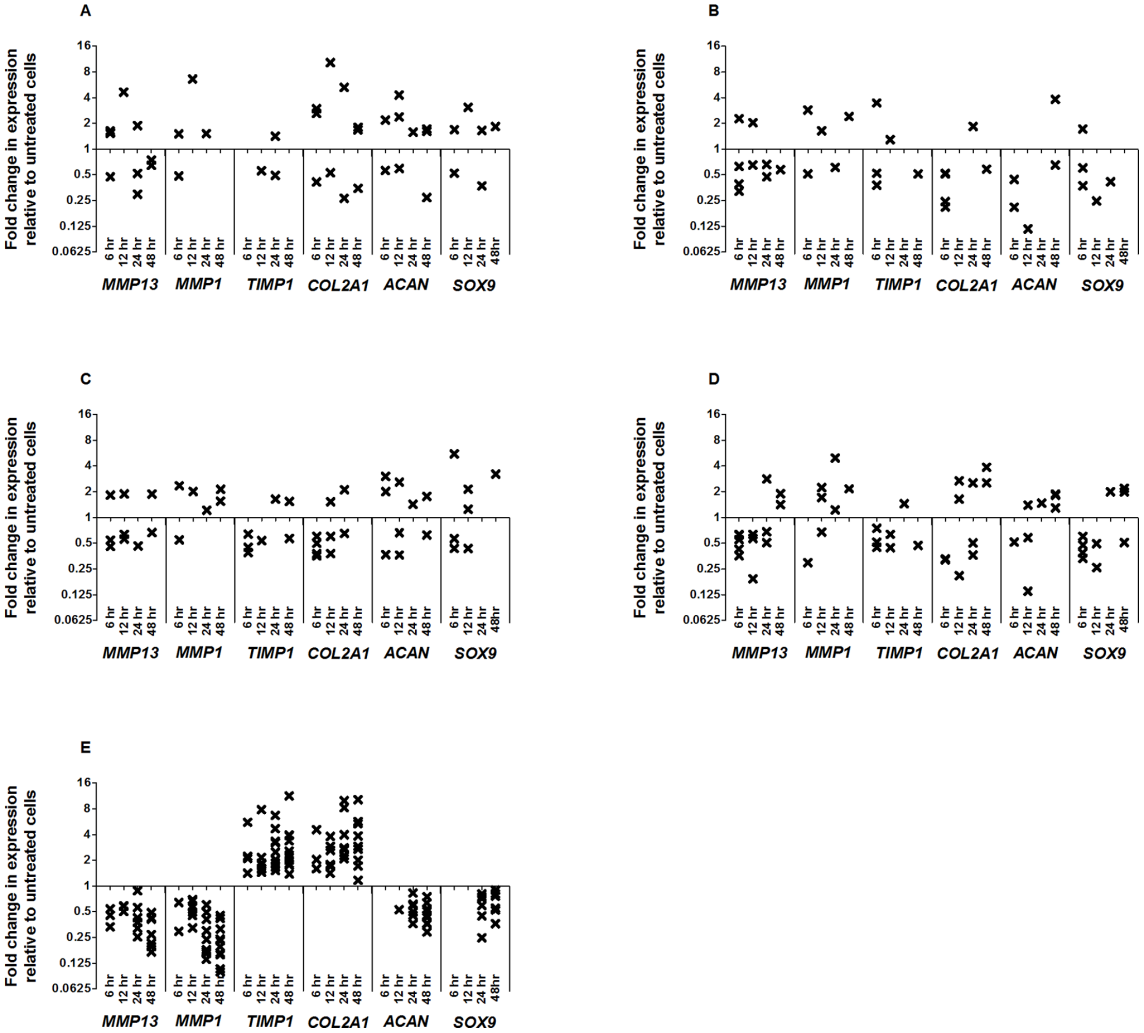
Gene expression changes in monolayer chondrocytes treated with GDF5 or TGF-β1 compared to untreated cells. Chondrocytes were stimulated for 6, 12, 24 and 48 hours with wildtype mouse GDF5 (A), wildtype human GDF5 (B), human GDF5 variant A (C), human GDF5 variant B (D) and TGF-β1 (E). Each cross represents a significant (P<0.05, two-tailed Student’s t-test) up/down regulation of gene expression relative to untreated cells in one patient. Twelve patients were studied for each of the GDF5 growth factor treatments and ten patients for the TGF-β1 treatments.

The cells were lysed at four time points after stimulation (6, 12, 24 and 48 hours). Changes in the expression of the six target genes *MMP13*, *MMP1*, *TIMP1*, *COL2A1*, *ACAN*, and *SOX9*, which code for proteins that have key catabolic and anabolic roles in chondrocyte biology, were then measured by qPCR. The relative gene expression of the test genes was compared between the treated and the untreated cells using a student’s two-tailed *t*-test. The significant (P<0.05) up/down changes in gene expression in response to stimulation with GDF5 proteins and with TGF-β1 are plotted in Figure 4. The actual values of these gene expression changes are listed in Tables S4–S8. A Wilcoxon signed rank test was performed to assess whether the chondrocytes showed a significant trend in response to each GDF5 and to TGF-β1. P-values are listed in Table 2.

**Table 2:** P-values calculated using the Wilcoxon signed rank test for each target gene following growth factor stimulation in monolayer culture.

Target gene	Growth factor				
	Wildtype mGDF5	Wildtype hGDF5	hGDF5 A	hGDF5 B	TGF-β1
*MMP13*	0.4849	0.0735	0.0598	0.1725	0.0002
*MMP1*	0.735	0.2884	0.6929	0.8697	<0.0001
*TIMP1*	0.2535	0.0561	0.5393	0.2827	<0.0001
*COL2A1*	0.4243	0.9755	0.2528	0.4823	<0.0001
*ACAN*	0.1236	0.0663	0.3942	0.9632	<0.0001
*SOX9*	0.7269	0.1961	0.2613	0.364	<0.0001

mGDF5, mouse GDF5; hGDF5, human GDF5. P<0.05 are highlighted in red.

Our results show that OA chondrocytes can respond to exogenous GDF5, with P-values greater than 0.05 for each of the six target genes analysed (Tables S4–S7), but not in a consistent manner. Some patients did not show a significant response to GDF5 stimulation at all (for example, patient 2), and where a response was observed at one time point, this did not necessarily persist through to further time points. For example, patient 9 showed a 1.64 fold significant upregulation of *MMP13*, 6 hours post stimulation with wildtype mouse GDF5, but with no change in *MMP13* expression at 12, 24 or 48 hours post stimulation (Table S4). Also, the changes in gene expression observed were not at all predictable, in that they were not always in the same direction. For example, *MMP13* was both up and down regulated in response to mouse, human and the variant A form of GDF5.

All ten patients cultured with TGF-β1 did however respond in a clearly consistent manner, showing an upregualtion of *TIMP1* (P<0.0001) and *COL2A1* (P<0.0001) and a down regulation of *MMP13* (P = 0.0002), *MMP1* (P<0.0001), *ACAN* (P<0.0001) and *SOX9* (P<0.0001; Figure 4E, Table 2 and Table S8).

We also harvested cells at 0 hours, prior to growth factor stimulation and were used for cDNA synthesis to measure the gene expression levels of the three GDF5 receptor genes (*BMPR-II*, *BMPR-IA* and *BMPR-IB*) and of the two TGF-β1 receptor genes (*TGFBR-II* and *TGFBR-I;* Figure S3). All of the patients studied demonstrated expression of the receptors, whilst for each receptor tested the levels of expression were comparable between patients. The lack of a consistent response following exogenous GDF5 treatment cannot therefore be due to an absence of receptor or to a relatively low level of receptor gene expression.

### None of the GDF5s Elicit a Consistent Target Gene Response by OA Chondrocytes in Micromass

Chondrocytes harvested from 19 OA patients (Table S2) were cultured with (treated) or without (untreated) 100 ng/ml of each of the GDF5 proteins. One of the 19 was cultured separately with wildtype human GDF5 and its variants (patient 21). Five of the 19 were cultured with wildtype mouse GDF5 and separately with 5 ng/ml of TGF-β1 (patients 22, 28–31). Another four were cultured with wildtype human GDF5 (patients 32–35). Four more were cultured with variant A and separately with variant B (patients 36–39). The remaining seven were cultured separately with wildtype mouse GDF5, wildtype human GDF5, variant A, variant B and with TGF-β1 (patients 23–27). As for the monolayer analysis, none of the culturing was performed with two or more of the growth factor proteins in the same culture mix.

The cells were lysed 5 days after stimulation, which is a time period sufficient for the cells to produce an extracellular matrix in culture, as determined by Alcian blue staining (data not shown). qPCR was used to measure any changes in the expression of the panel of six target genes. The relative gene expression of the target genes was compared between the treated and the untreated cells by performing a student’s two-tailed *t*-test.

**Figure 5:**
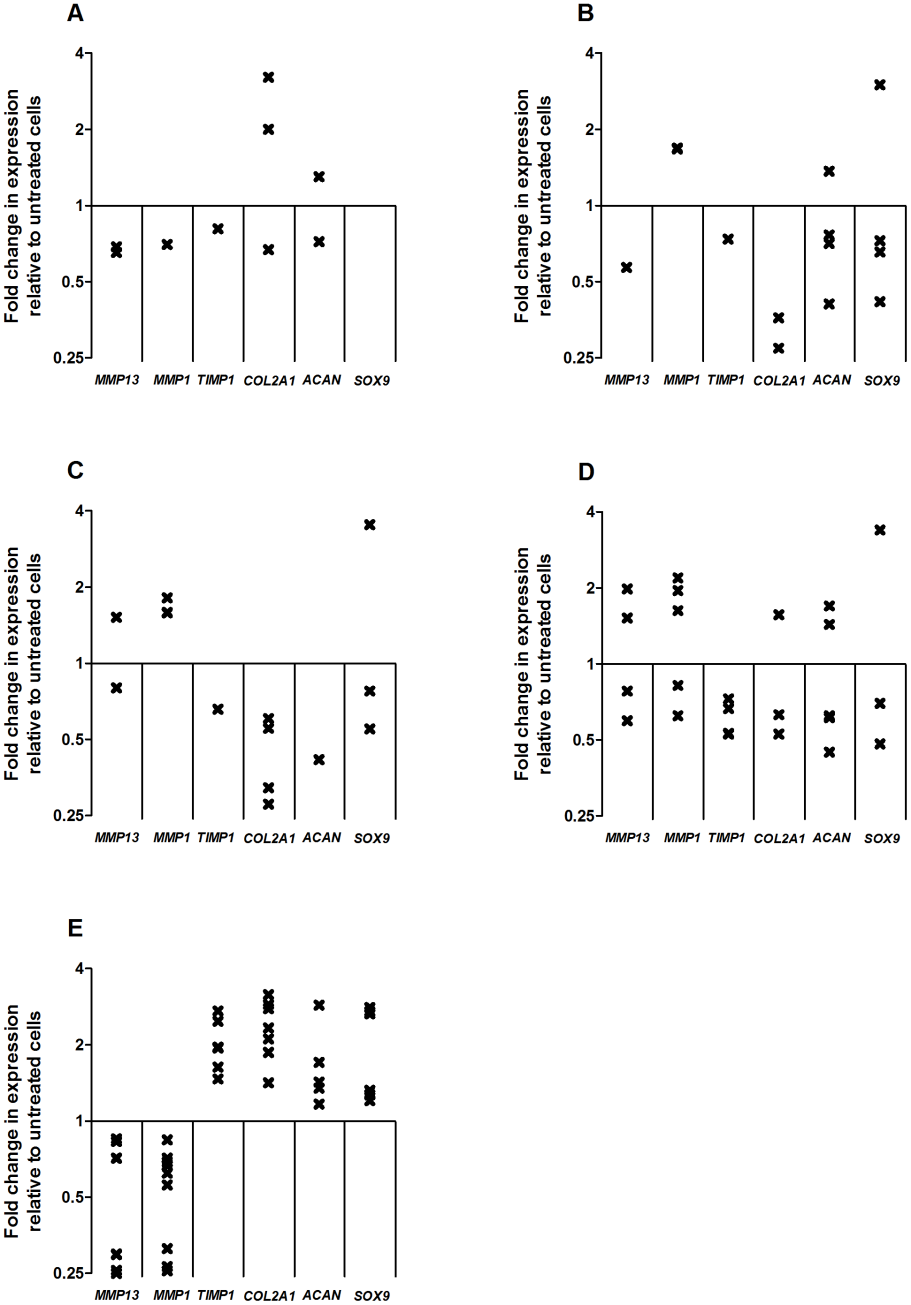
Gene expression changes in micromass chondrocytes treated with GDF5 or TGF-β1 compared to untreated cells. Chondrocytes were stimulated for 5 days with wildtype mouse GDF5 (A), wildtype human GDF5 (B), human GDF5 variant A (C), human GDF5 variant B (D) and TGF-β1 (E). Each cross represents a significant (P<0.05, two-tailed Student’s t-test) up/down regulation of gene expression relative to untreated cells in one patient. Ten patients were studied for each of the growth factor treatments.

The significant (P<0.05) up/down changes in gene expression in response to stimulation with GDF5 proteins and with TGF-β1 are plotted in Figure 5. The actual values of these gene expression changes are listed in Tables S9–S13. A Wilcoxon signed rank test was performed to assess whether the chondrocytes showed a significant trend in response to each GDF5 and to TGF-β1. P-values are listed in Table 3.

**Table 3:** P-values calculated using the Wilcoxon signed rank test for each target gene following growth factor stimulation in micromasss culture.

Target gene	Growth factor				
	Wildtype mGDF5	Wildtype hGDF5	hGDF5 A	hGDF5 B	TGF-β1
*MMP13*	0.1027	0.5074	0.7596	0.9188	0.0090
*MMP1*	0.6953	0.1055	0.2210	0.1934	0.0039
*TIMP1*	0.3326	0.0645	1.0000	0.0829	0.0020
*COL2A1*	0.2324	1.0000	0.2324	0.6250	0.0020
*ACAN*	0.4065	0.8203	0.5566	0.6101	0.0020
*SOX9*	0.8383	0.1055	0.4316	0.5566	0.0091

mGDF5, mouse GDF5; hGDF5, human GDF5. P<0.05 are highlighted in red.

As for the monolayer analysis, there was a limited and discordant response of the micromasses to GDF5, with any change in gene expression often not in the same direction. For example, in response to human GDF5 variant B, patients 24 and 38 showed a 0.78 and 0.69 fold significant down regulation of *MMP13* whereas patients 26 and 27 showed a 1.98 and 1.52 fold significant upregulation of this gene.

However, and was observed for monolayer analysis, the patients cultured with TGF-β1 did respond in a clearly consistent manner (Table S13 and Figure 5E). TGF-β1resulted in a down regulation of *MMP13* (P = 0.0090) and *MMP1* (P = 0.0039) and an upregulation of *TIMP1* (P = 0.0020) and *COL2A1* (P = 0.0020), *SOX9* (P = 0.0091) and *ACAN* (P = 0.002). The direction of the responses for *MMP13*, *MMP1*, *TIMP1* and *COL2A1* were the same as those seen for the monolayer chondrocytes. However, the upregulation observed for *SOX9* and *ACAN* in micromass culture was opposite to what was observed in monolayer. This probably reflects differences between the two culture conditions [28].

We measured the expression of GDF5 and TGF-β1 receptor genes in chondrocytes from patients 21–39 after 5 days of culture in micromass without growth factor stimulation (Figure S4). As in the monolayer analysis (Figure S3) all of the patients demonstrated expression of the receptors, whilst for each receptor tested the levels of expression were comparable between the patients. There was therefore no link between receptor gene expression and response to growth factor stimulation.

## Discussion

We initially demonstrated the expression of all three GDF5 receptor genes in RNA extracted directly from cartilage chondrocytes and in RNA derived from the chondrocyte monolayer and micromass cultures. In all three cases *BMPR-IB*, which encodes the type I receptor that GDF5 preferentially binds to, showed a lower level of expression relative to *BMPR-II* and *BMPR-IA*. Western blot analysis confirmed lower levels of the BMPR-IB protein. Others have also previously noted a difference in the expression of the proteins encoded by these genes in OA cartilage [29]. We therefore chose to study, in addition to wildtype mouse and human GDF5, a variant form of human GDF5 that was designed by Biopharm to preferentially bind to BMPR-IA. Also, we included in our study another variant form of GDF5 that was designed by Biopharm to be insensitive to the GDF5 antagonist noggin.

Each of the four forms of GDF5 increased luciferase activity of a Smad responsive vector and they also phosphorylated Smad 1/5/8, confirming that they were able to bring about translocation of a growth factor signal. They were also able to stimulate a change in the expression of a panel of target genes in both monolayer and micromass chondrocytes. However, and unlike TGF-β1, the response of chondrocytes was not at all consistent for any of the four different forms of GDF5. The response to TGF-β1 demonstrates that chondrocytes from OA patients are clearly capable of responding to growth factors of the TGF-β superfamily in a predictable manner; the inconsistency with regards to GDF5 is not therefore a systemic characteristic of these cells. We also measured the gene expression levels of GDF5 and TGF-β1 receptor genes in chondrocytes from each patient and did not find any link between their response to exogenous growth factors and the their receptor gene expression levels. However, it is possible that the GDF5 receptor expression levels may change during the course of the experiment, which may account for some of the inconsistencies in response seen between time points and among patients. Also, the dose of exogenous GDF5 that we used may be suboptimal or inhibitory for some samples, and may generate inconsistent results between patients.

Guerne *et al*. [30] have shown a reduction in proliferative response with increasing age in chondrocytes in response to the growth factors PDGF-AA, FGF-2, IGF-1 and TGF-β1. Interestingly, the authors observed that TGF-β1 was the most potent stimulant analysed, and was the only factor that consistently and significantly increased the proliferation rate in chondrocytes from older donors. Loeser *et al*. [31] have also reported on a reduction in the chondrocyte response to IGF-1 with increasing age and histologic OA score in cynomolgus monkeys that have naturally occurring OA. However, our principal observation was that where there was a response to GDF5, this response was inconsistent between the chondrocytes of OA patients.

The association of the *GDF5* SNP rs143383 with OA is one of the most robust reported to-date, with replication observed in Asians and Europeans, despite the different polymorphic architecture of the gene between these two ethnics groups, and with a variety of supporting functional data, much of which directly targets the SNP itself (reviewed in [32]). The prevailing data therefore strongly supports further investigation of this gene and its protein in the context of OA.

Whether the discordant response of chondrocytes to GDF5 that we have observed is a cause or a consequence of the OA disease process we cannot say. A loss of consistent response to growth factors during ageing may contribute to the development and progression of OA, resulting in an altered balance between anabolism and catabolism in some patients. Hence, it is important to develop a means to enhance the responsive capacity of chondrocytes if GDF5 can potentially be used as a therapeutic to overcome the genetic deficit mediated by the OA risk allele at rs143383.

## Supporting Information

Figure S1 **GDF5 receptor protein expression in cultured chondrocytes.** The figure is representative of three separate experiments.
(DOCX)

Figure S2 **GDF5 receptor gene expression in SW1353 chondrosarcoma cells.** Gene expression was measured relative to the housekeepers, *18S*, *GAPDH* and *HPRT1*. Five technical replicates were performed for each gene. The error bars represent the standard error of the mean.
(DOCX)

Figure S3 **GDF5 and TGF-β1 receptor gene expression.** Gene expression of *BMPR-II* (A), *BMPR-IA* (B) and *BMPR-IB* (C) was measured in chondrocytes from 25 patients that were cultured in monolayer prior to stimulation with exogenous GDF5. Gene expression of *TGFBR-II* (D) and *TGFBR-I* (E) was measured in chondrocytes from 10 patients that were cultured in monolayer prior to stimulation with exogenous TGF-β1. Gene expression was measured relative to the housekeepers, *18S*, *GAPDH* and *HPRT1*. The error bars represent the standard error of the mean of six biological replicates. Dotted lines represent the average gene expression across all patients studied.
(DOCX)

Figure S4 **GDF5 and TGF-β1 receptor gene expression.** Gene expression of BMPR-II (A), BMPR-IA (B) and BMPR-IB (C) was measured in untreated chondrocytes from 19 patients that were cultured in micromass which were used to analyse the response to exogenous GDF5. Gene expression of *TGFBR-II* (D) and *TGFBR-I* (E) was measured in chondrocytes from 10 patients that were cultured in micromass which were used to analyse the response to exogenous TGF-β1. Gene expression was measured relative to the housekeepers, *18S*, *GAPDH* and *HPRT1*. The error bars represent the standard error of the mean of four biological replicates. Dotted lines represent the average gene expression across all patients studied.
(DOCX)

Table S1 **Details of the OA patients and of the growth factors used to stimulate their chondrocytes in the monolayer culture experiment.** F, female; M, male; mGDF5, mouse GDF5; hGDF5, human GDF5.
(DOCX)

Table S2 **Details of the OA patients and of the growth factors used to stimulate their chondrocytes in the micromass culture experiment.** F, female; M, male; mGDF5, mouse GDF5; hGDF5, human.
(DOCX)

Table S3 **Primer and probe sequences for the TaqMan assays used for quantitative real time PCR.**
(DOCX)

Table S4 **The changes in expression of the target genes following OA chondrocyte monolayer culturing and stimulation with wildtype mouse GDF5.** The chondrocytes from twelve OA patients were cultured with or without wildtype mouse GDF5 and gene expression was measured at 6, 12, 24 and 48 hours post stimulation. The actual values of any significant (P≤0.05, two-tailed Student’s t-test) fold changes in expression of the six target genes in response to the stimulation are shown in bold text. A value greater than 1 denotes an up regulation of gene expression and a value less than 1 denotes a down regulation of gene expression.
(DOCX)

Table S5 **The changes in expression of the target genes following OA chondrocyte monolayer culturing and stimulation with wildtype human GDF5.** The chondrocytes from twelve OA patients were cultured with or without wildtype human GDF5 and gene expression was measured at 6, 12, 24 and 48 hours post stimulation. The actual values of any significant (P≤0.05, two-tailed Student’s t-test) fold changes in expression of the six target genes in response to the stimulation are shown in bold text. A value greater than 1 denotes an up regulation of gene expression and a value less than 1 denotes a down regulation of gene expression.
(DOCX)

Table S6 **The changes in expression of the target genes following OA chondrocyte monolayer culturing and stimulation with human GDF5 variant A.** The chondrocytes from twelve OA patients were cultured with or without variant A and gene expression was measured at 6, 12, 24 and 48 hours post stimulation. The actual values of any significant (P≤0.05, two-tailed Student’s t-test) fold changes in expression of the six target genes in response to the stimulation are shown in bold text. A value greater than 1 denotes an up regulation of gene expression and a value less than 1 denotes a down regulation of gene expression.
(DOCX)

Table S7 **The changes in expression of the target genes following OA chondrocyte monolayer culturing and stimulation with human GDF5 variant B.** The chondrocytes from twelve OA patients were cultured with or without variant B and gene expression was measured at 6, 12, 24 and 48 hours post stimulation. The actual values of any significant (P≤0.05, two-tailed Student’s t-test) fold changes in expression of the six target genes in response to the stimulation are shown in bold text. A value greater than 1 denotes an up regulation of gene expression and a value less than 1 denotes a down regulation of gene expression.
(DOCX)

Table S8 **The changes in expression of the target genes following OA chondrocyte monolayer culturing and stimulation with TGF-β1.** The chondrocytes from ten OA patients were cultured with or without TGF-β1 and gene expression was measured at 6, 12, 24 and 48 hours post stimulation. The actual values of any significant (P≤0.05, two-tailed Student’s t-test) fold changes in expression of the six target genes in response to the stimulation are shown in bold text. A value greater than 1 denotes an up regulation of gene expression and a value less than 1 denotes a down regulation of gene expression.
(DOCX)

Table S9 **The changes in expression of the target genes following OA chondrocyte micromass culturing and stimulation with wildtype mouse GDF5.** The chondrocytes from ten OA patients were cultured with or without wildtype mouse GDF5 and gene expression was measured 5 days post stimulation. The actual values of any significant (P≤0.05, two-tailed Student’s t-test) fold changes in expression of the six target genes in response to the stimulation are shown in bold text. A value greater than 1 denotes an up regulation of gene expression and a value less than 1 denotes a down regulation of gene expression.
(DOCX)

Table S10 **The changes in expression of the target genes following OA chondrocyte micromass culturing and stimulation with wildtype human GDF5.** The chondrocytes from ten OA patients were cultured with or without wildtype human GDF5 and gene expression was measured 5 days post stimulation. The actual values of any significant (P≤0.05, two-tailed Student’s t-test) fold changes in expression of the six target genes in response to the stimulation are shown in bold text. A value greater than 1 denotes an up regulation of gene expression and a value less than 1 denotes a down regulation of gene expression.
(DOCX)

Table S11 **The changes in expression of the target genes following OA chondrocyte micromass culturing and stimulation with human GDF5 variant A.** The chondrocytes from ten OA patients were cultured with or without variant A and gene expression was measured 5 days post stimulation. The actual values of any significant (P≤0.05, two-tailed Student’s t-test) fold changes in expression of the six target genes in response to the stimulation are shown in bold text. A value greater than 1 denotes an up regulation of gene expression and a value less than 1 denotes a down regulation of gene expression.
(DOCX)

Table S12 **The changes in expression of the target genes following OA chondrocyte micromass culturing and stimulation with human GDF5 variant B.** The chondrocytes from ten OA patients were cultured with or without variant B and gene expression was measured 5 days post stimulation. The actual values of any significant (P≤0.05, two-tailed Student’s t-test) fold changes in expression of the six target genes in response to the stimulation are shown in bold text. A value greater than 1 denotes an up regulation of gene expression and a value less than 1 denotes a down regulation of gene expression.
(DOCX)

Table S13 **The changes in expression of the target genes following OA chondrocyte micromass culturing and stimulation with TGF-β1.** The chondrocytes from ten OA patients were cultured with or without TGF-β1 and gene expression was measured 5 days post stimulation. The actual values of any significant (P≤0.05, two-tailed Student’s t-test) fold changes in expression of the six target genes in response to the stimulation are shown in bold text. A value greater than 1 denotes an up regulation of gene expression and a value less than 1 denotes a down regulation of gene expression.
(DOCX)
