# Supplementary material for: Human Chondrocytes Respond Discordantly to the Protein Encoded by the Osteoarthritis Susceptibility Gene GDF5
Source: PLoS One. 2014 Jan 21;9(1):e86590. doi: 10.1371/journal.pone.0086590 (PMC3897745; doi:10.1371/journal.pone.0086590)

**Figure S1.** GDF5 receptor protein expression in cultured chondrocytes.

This data comes from one OA patient. Identical data was obtained for two more OA patients. (data not shown).


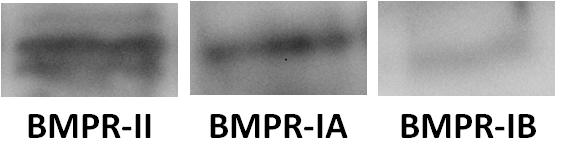

Supplement: Figure S1 — GDF5 receptor protein expression in cultured chondrocytes. The figure is representative of three separate experiments. (DOCX) [file pone.0086590.s001.docx]
