# Supplementary material for: Human Chondrocytes Respond Discordantly to the Protein Encoded by the Osteoarthritis Susceptibility Gene GDF5
Source: PLoS One. 2014 Jan 21;9(1):e86590. doi: 10.1371/journal.pone.0086590 (PMC3897745; doi:10.1371/journal.pone.0086590)

**Figure S2.** GDF5 receptor gene expression in SW1353 chondrosarcoma cells.

Gene expression was measured relative to the housekeepers, *18S*, *GAPDH* and *HPRT1*. Five technical replicates were performed for each gene. The error bars represent the standard error of the mean.


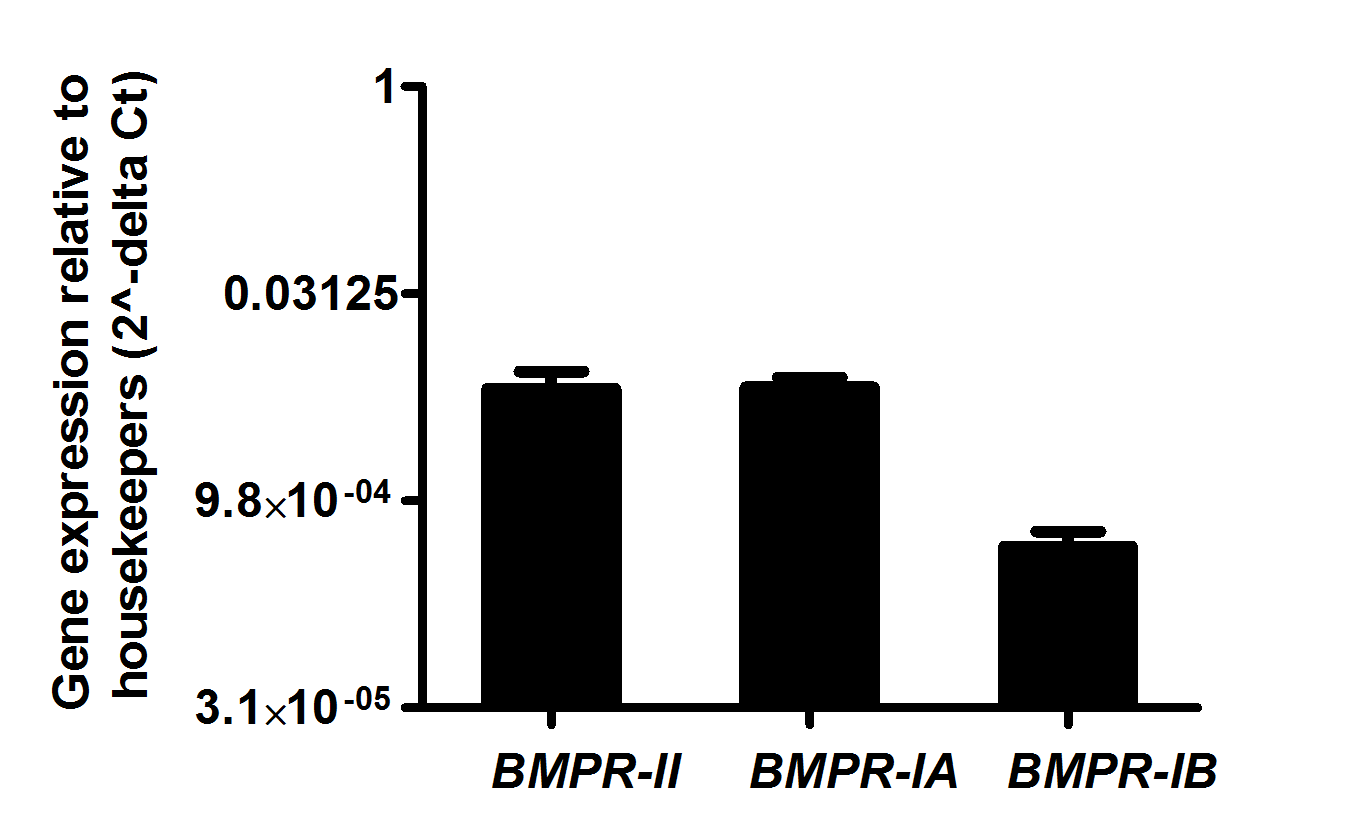

Supplement: Figure S2 — GDF5 receptor gene expression in SW1353 chondrosarcoma cells. Gene expression was measured relative to the housekeepers, 18S, GAPDH and HPRT1. Five technical replicates were performed for each gene. The error bars represent the standard error of the mean. (DOCX) [file pone.0086590.s002.docx]
