# Supplementary material for: Human Chondrocytes Respond Discordantly to the Protein Encoded by the Osteoarthritis Susceptibility Gene GDF5
Source: PLoS One. 2014 Jan 21;9(1):e86590. doi: 10.1371/journal.pone.0086590 (PMC3897745; doi:10.1371/journal.pone.0086590)

**Figure S3.** GDF5 and TGF-β1 receptor gene expression.

Gene expression of *BMPR-II* (A), *BMPR-IA* (B) and *BMPR-IB* (C) was measured in chondrocytes from 25 patients that were cultured in monolayer prior to stimulation with exogenous GDF5. Gene expression of *TGFBR-II* (D) and *TGFBR-I* (E) was measured in chondrocytes from 10 patients that were cultured in monolayer prior to stimulation with exogenous TGF-β1. Gene expression was measured relative to the housekeepers, *18S*, *GAPDH* and *HPRT1*. The error bars represent the standard error of the mean of six biological replicates. Dotted lines represent the average gene expression across all patients studied.


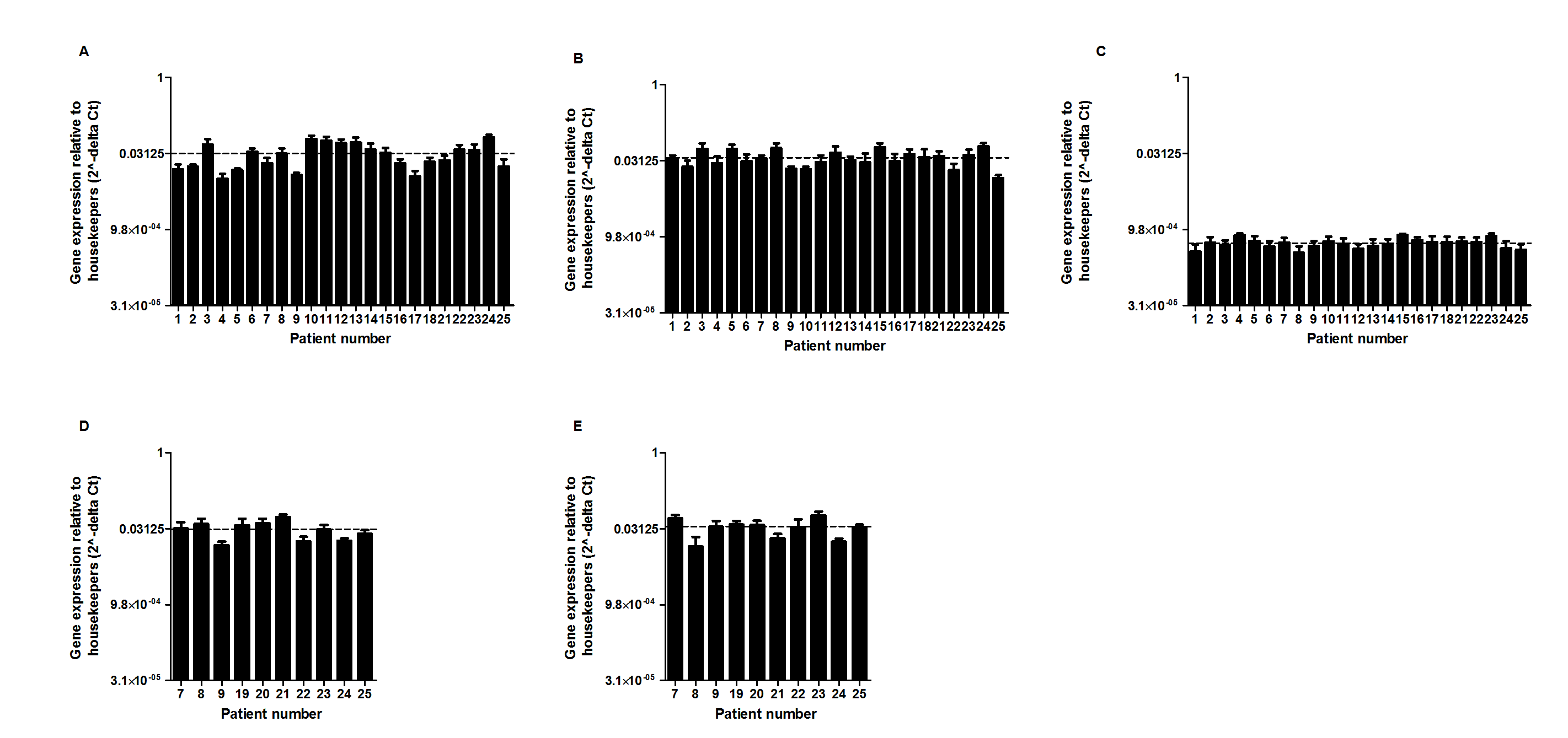

Supplement: Figure S3 — GDF5 and TGF-β1 receptor gene expression. Gene expression of BMPR-II (A), BMPR-IA (B) and BMPR-IB (C) was measured in chondrocytes from 25 patients that were cultured in monolayer prior to stimulation with exogenous GDF5. Gene expression of TGFBR-II (D) and TGFBR-I (E) was measured in chondrocytes from 10 patients that were cultured in monolayer prior to stimulation with exogenous TGF-β1. Gene expression was measured relative to the housekeepers, 18S, GAPDH and HPRT1. The error bars represent the standard error of the mean of six biological replicates. Dotted lines represent the average gene expression across all patients studied. (DOCX) [file pone.0086590.s003.docx]
