# Supplementary material for: Human Chondrocytes Respond Discordantly to the Protein Encoded by the Osteoarthritis Susceptibility Gene GDF5
Source: PLoS One. 2014 Jan 21;9(1):e86590. doi: 10.1371/journal.pone.0086590 (PMC3897745; doi:10.1371/journal.pone.0086590)
Supplement: Table S1 — Details of the OA patients and of the growth factors used to stimulate their chondrocytes in the monolayer culture experiment. F, female; M, male; mGDF5, mouse GDF5; hGDF5, human GDF5. (DOCX) [file pone.0086590.s005.docx]

**Table S1.** Details of the OA patients and of the growth factors used to stimulate their chondrocytes in the monolayer culture experiment.

| **Patient number** | **Age in years at surgery** | **Sex** | **Joint replaced** | **Growth factor** |
| --- | --- | --- | --- | --- |
| 1 | 59 | M | Knee | Wildtype mGDF5 |
| 2 | 70 | M | Knee |  |
| 3 | 68 | F | Knee |  |
| 4 | 45 | F | Knee |  |
| 5 | 75 | M | Knee |  |
| 6 | 46 | M | Knee |  |
| 7 | 73 | F | Knee | Wildtype mGDF5, TGF-β1 |
| 8 | 77 | M | Knee |  |
| 9 | 68 | M | Knee |  |
| 10 | 66 | M | Hip | Wildtype hGDF5 |
| 11 | 64 | F | Knee |  |
| 12 | 63 | M | Knee | hGDF5 variant A, hGDF5 variant B |
| 13 | 78 | F | Knee |  |
| 14 | 84 | M | Hip | Wildtype hGDF5, hGDF5 variant A, hGDF5 variant B |
| 15 | 82 | M | Knee |  |
| 16 | 70 | F | Knee |  |
| 17 | 70 | F | Knee |  |
| 18 | 68 | F | Knee |  |
| 19 | 67 | F | Knee | TGF-β1 |
| 20 | 58 | F | Knee |  |
| 21 | 82 | F | Knee | Wildtype mGDF5, Wildtype hGDF5, hGDF5 variant A, hGDF5 variant B, TGF-β1 |
| 22 | 60 | M | Knee |  |
| 23 | 58 | F | Knee |  |
| 24 | 59 | M | Knee | Wildtype hGDF5, hGDF5 variant A, hGDF5 variant B, TGF-β1 |
| 25 | 54 | M | Knee |  |

F, female; M, male; mGDF5, mouse GDF5; hGDF5, human GDF5
