# Supplementary material for: Human Chondrocytes Respond Discordantly to the Protein Encoded by the Osteoarthritis Susceptibility Gene GDF5
Source: PLoS One. 2014 Jan 21;9(1):e86590. doi: 10.1371/journal.pone.0086590 (PMC3897745; doi:10.1371/journal.pone.0086590)
Supplement: Table S2 — Details of the OA patients and of the growth factors used to stimulate their chondrocytes in the micromass culture experiment. F, female; M, male; mGDF5, mouse GDF5; hGDF5, human. (DOCX) [file pone.0086590.s006.docx]

**Table S2.** Details of the OA patients and of the growth factors used to stimulate their chondrocytes in the micromass culture experiment.

| **Patient number** | **Age in years at surgery** | **Sex** | **Joint replaced** | **Growth factor** |
| --- | --- | --- | --- | --- |
| 21 | 82 | F | Knee | Wildtype hGDF5, hGDF5 variant A, hGDF5 variant B |
| 22 | 60 | M | Knee | Wildtype mGDF5, TGF-β1 |
| 23 | 58 | F | Knee | Wildtype mGDF5, Wildtype hGDF5, hGDF5 variant A, hGDF5 variant B, TGF-β1 |
| 24 | 59 | M | Knee |  |
| 25 | 54 | M | Knee |  |
| 26 | 55 | M | Knee |  |
| 27 | 49 | F | Knee |  |
| 28 | 69 | M | Knee | Wildtype mGDF5, TGF-β1 |
| 29 | 62 | F | Knee |  |
| 30 | 54 | F | Knee |  |
| 31 | 66 | F | Knee |  |
| 32 | 69 | F | Knee | Wildtype hGDF5 |
| 33 | 45 | M | Hip |  |
| 34 | 61 | F | Hip |  |
| 35 | 62 | F | Knee |  |
| 36 | 65 | M | Knee | hGDF5 variant A, hGDF5 variant B |
| 37 | 80 | F | Knee |  |
| 38 | 57 | M | Knee |  |
| 39 | 79 | F | Knee |  |

F, female; M, male; mGDF5, mouse GDF5; hGDF5, human
