# Supplementary material for: Human Chondrocytes Respond Discordantly to the Protein Encoded by the Osteoarthritis Susceptibility Gene GDF5
Source: PLoS One. 2014 Jan 21;9(1):e86590. doi: 10.1371/journal.pone.0086590 (PMC3897745; doi:10.1371/journal.pone.0086590)
Supplement: Table S3 — Primer and probe sequences for the TaqMan assays used for quantitative real time PCR. (DOCX) [file pone.0086590.s007.docx]

**Table S3.** Primer and probe sequences for the TaqMan assays used for quantitative real time PCR.

| **Gene** | **Forward primer (5'-3')** | **Reverse primer (5'-3')** | **Probe (5'-3')** |
| --- | --- | --- | --- |
| *GAPDH* | ACATCGCTCAGACACCATG | TGTAGTTGAGGTCAATGAAGGG | AAGGTCGGAGTCAACGGATTTGGTC |
| *HPRT1* | TGCTGAGGATTTGGAAAGGG | ACAGAGGGCTACAATGTGATG | AGGACTGAACGTCTTGCTCGAGATG |
| *18s* | CGAATGGCTCATTAAATCAGTTATGG | TATTAGCTCTAGAATTACCACAGTTATCC | TCCTTTGGTCGCTCGCTCCTCTCCC |
| *MMP13* | AAATTATGGAGGAGATGCCCATT | TCCTTGGAGTGGTCAAGACCTAA | CTACAACTTGTTTCTTGTTGCTGCGCATGA |
| *MMP1* | AAGATGAAAGGTGGACCAACAATT | CCAAGAGAATGGCCGAGTTC | CAGAGAGTACAACTTACATCGTGTTGCGGCTC |
| *TIMP1* | TTCTGCAATTCCGACCTCG | TCATAACGCTGGTATAAGGTGG | TTGACTTCTGGTGTCCCCACGAAC |
| *COL2A1* | ACCTTCATGGCGTCCAAG | AACCAGATTGAGAGCATCCG | AGACCTGAAACTCTGCCACCCTG |
| *SOX9* | CTGGTACTTGTAATCCGGGTG | ACTTGCACAACGCCGAG | TCTGGAGACTTCTGAACGAGAGCGA |
| *ACAN* | TGTGGGACTGAAGTTCTTGG | AGCGAGTTGTCATGGTCTG | CTGGGTTTTCGTGACTCTGAGGGT |
| *BMPRII* | GGCTGACTGGAAATAGACTGG | CACAGTCCCTCAAGTTCACAG | CCTCGCTTATGGCTGCATTATCTTCCTC |
| *BMPRIA* | ACAAAGTTCTGGTAGTGGGTC | CATCCATACTTCTCCATATCGGC | ATTCAGATGGTCCGGCAAGTTGGT |
| *BMPRIB* | CTGCACAGAAAGGAACGAATG | AGGACCAAGAGCAAACTACAG | TGGAGGCAGTGTAGGGTGTAGGT |
