# Supplementary material for: Human Chondrocytes Respond Discordantly to the Protein Encoded by the Osteoarthritis Susceptibility Gene GDF5
Source: PLoS One. 2014 Jan 21;9(1):e86590. doi: 10.1371/journal.pone.0086590 (PMC3897745; doi:10.1371/journal.pone.0086590)
Supplement: Table S5 — The changes in expression of the target genes following OA chondrocyte monolayer culturing and stimulation with wildtype human GDF5. The chondrocytes from twelve OA patients were cultured with or without wildtype human GDF5 and gene expression was measured at 6, 12, 24 and 48 hours post stimulation. The actual values of any significant (P≤0.05, two-tailed Student’s t-test) fold changes in expression of the six target genes in response to the stimulation are shown in bold text. A value greater than 1 denotes an up regulation of gene expression and a value less than 1 denotes a down regulation of gene expression. (DOCX) [file pone.0086590.s009.docx]

Table S5. The changes in expression of the target genes following OA chondrocyte monolayer culturing and stimulation with wildtype human GDF5. The chondrocytes from twelve OA patients were cultured with or without wildtype human GDF5 and gene expression was measured at 6, 12, 24 and 48 hours post stimulation. The actual values of any significant (P≤0.05, two-tailed Student’s t-test) fold changes in expression of the six target genes in response to the stimulation are shown in bold text. A value greater than 1 denotes an up regulation of gene expression and a value less than 1 denotes a down regulation of gene expression.

| **Gene** | **Time point after stimulation** | **Patient** | | | | | | | | | | | |
| --- | --- | --- | --- | --- | --- | --- | --- | --- | --- | --- | --- | --- | --- |
|  |  | **10** | **11** | **14** | **15** | **16** | **17** | **18** | **21** | **22** | **23** | **24** | **25** |
| ***MMP13*** | 6 hours | 0.57 | **0.39** | 0.79 | 0.65 | 0.38 | **2.27** | 0.57 | **0.32** | 0.74 | 1.27 | 1.01 | **0.63** |
|  | 12 hours | 0.16 | **0.65** | 1.28 | 0.46 | 1.78 | 1.51 | 0.83 | 0.55 | 0.68 | 1.47 | **2.03** | 1.18 |
|  | 24 hours | 1.26 | 0.98 | 1.15 | 1.04 | 1.18 | 0.47 | 0.70 | 0.62 | 0.76 | 0.76 | **0.48** | **0.66** |
|  | 48 hours | 2.36 | 0.86 | 0.50 | 1.05 | 0.83 | **0.58** | 0.85 | 0.93 | 0.80 | 1.17 | 1.33 | 1.28 |
| ***MMP1*** | 6 hours | 1.26 | 0.65 | 0.57 | **2.87** | 0.45 | 0.92 | 0.73 | 0.56 | 0.78 | 1.20 | 1.31 | **0.51** |
|  | 12 hours | 0.24 | 0.76 | 0.83 | 0.44 | 3.22 | 1.10 | 0.83 | 0.86 | 0.14 | 1.45 | 1.60 | **1.64** |
|  | 24 hours | 1.19 | 0.98 | 0.49 | 0.42 | 0.67 | 0.36 | 0.90 | 0.80 | 0.87 | 0.85 | 0.91 | **0.61** |
|  | 48 hours | 0.65 | 0.77 | 1.02 | 0.91 | 0.79 | 0.77 | 0.98 | 6.95 | 1.16 | 1.88 | 3.18 | **2.42** |
| ***TIMP1*** | 6 hours | 1.30 | **0.52** | 0.56 | **3.46** | 0.68 | 1.26 | 0.77 | **0.38** | 0.98 | 0.82 | 0.97 | 0.82 |
|  | 12 hours | 0.31 | 0.84 | 1.08 | 0.68 | 1.89 | 1.29 | 0.96 | 1.07 | 0.41 | 1.13 | **1.29** | 0.94 |
|  | 24 hours | 0.90 | 0.80 | 0.79 | 0.73 | 0.72 | 0.47 | 1.15 | 0.51 | 1.28 | 0.72 | 0.95 | 1.00 |
|  | 48 hours | 0.31 | 0.69 | 1.14 | 0.83 | 1.12 | 0.70 | 0.98 | 2.89 | 0.73 | 1.51 | 1.04 | **0.51** |
| ***COL2A1*** | 6 hours | 1.46 | **0.52** | 1.35 | 0.46 | **0.21** | 1.26 | 1.26 | **0.24** | **0.51** | 0.50 | 0.73 | 0.85 |
|  | 12 hours | 0.15 | 0.78 | 1.17 | 0.66 | 1.23 | 1.67 | 0.90 | 1.59 | 0.34 | 2.28 | 1.14 | 1.18 |
|  | 24 hours | 1.19 | 0.81 | 1.83 | 0.91 | 1.24 | 0.51 | 0.78 | 2.15 | 1.01 | 0.60 | 1.01 | **1.84** |
|  | 48 hours | 1.38 | 2.49 | 2.35 | 1.05 | 0.81 | 0.77 | 0.85 | 0.00 | 1.89 | 0.56 | 1.11 | **0.58** |
| ***ACAN*** | 6 hours | 0.85 | **0.44** | 0.94 | 0.78 | **0.21** | 1.36 | 1.02 | 0.70 | 0.68 | 0.68 | 0.95 | 0.76 |
|  | 12 hours | 0.24 | 0.70 | 0.98 | 0.60 | 0.93 | 1.44 | 0.94 | 1.38 | **0.12** | 0.77 | 1.19 | 1.05 |
|  | 24 hours | 1.32 | 0.76 | 0.55 | 0.46 | 1.04 | 0.63 | 1.15 | 1.24 | 0.79 | 0.74 | 0.94 | 1.03 |
|  | 48 hours | 0.45 | 1.65 | 2.03 | 0.94 | **3.80** | **0.65** | 0.98 | 1.06 | 1.24 | 1.36 | 0.70 | 0.68 |
| ***SOX9*** | 6 hours | 1.43 | 0.77 | **1.72** | 1.18 | **0.37** | 1.05 | 0.97 | 0.49 | 0.73 | 1.05 | 1.24 | **0.60** |
|  | 12 hours | 0.11 | 0.93 | 1.04 | 0.74 | 1.17 | 0.97 | 0.90 | 0.83 | **0.25** | 0.96 | 1.10 | 1.12 |
|  | 24 hours | 0.90 | 0.94 | 0.90 | 0.37 | 0.55 | 0.55 | 0.77 | **0.42** | 1.03 | 0.54 | 0.97 | 1.06 |
|  | 48 hours | 1.38 | 0.90 | 2.01 | 0.70 | 2.21 | 0.76 | 0.86 | 1.21 | 1.03 | 1.37 | 1.55 | 1.08 |
