# Supplementary material for: Human Chondrocytes Respond Discordantly to the Protein Encoded by the Osteoarthritis Susceptibility Gene GDF5
Source: PLoS One. 2014 Jan 21;9(1):e86590. doi: 10.1371/journal.pone.0086590 (PMC3897745; doi:10.1371/journal.pone.0086590)
Supplement: Table S8 — The changes in expression of the target genes following OA chondrocyte monolayer culturing and stimulation with TGF-β1. The chondrocytes from ten OA patients were cultured with or without TGF-β1 and gene expression was measured at 6, 12, 24 and 48 hours post stimulation. The actual values of any significant (P≤0.05, two-tailed Student’s t-test) fold changes in expression of the six target genes in response to the stimulation are shown in bold text. A value greater than 1 denotes an up regulation of gene expression and a value less than 1 denotes a down regulation of gene expression. (DOCX) [file pone.0086590.s012.docx]

Table S8. The changes in expression of the target genes following OA chondrocyte monolayer culturing and stimulation with TGF-β1. The chondrocytes from ten OA patients were cultured with or without TGF-β1 and gene expression was measured at 6, 12, 24 and 48 hours post stimulation. The actual values of any significant (P≤0.05, two-tailed Student’s t-test) fold changes in expression of the six target genes in response to the stimulation are shown in bold text. A value greater than 1 denotes an up regulation of gene expression and a value less than 1 denotes a down regulation of gene expression.

| **Gene** | **Time point after stimulation** | **Patient** | | | | | | | | | |
| --- | --- | --- | --- | --- | --- | --- | --- | --- | --- | --- | --- |
|  |  | **7** | **8** | **9** | **19** | **20** | **21** | **22** | **23** | **24** | **25** |
| ***MMP13*** | 6 hours | 1.04 | 0.89 | 1.30 | **0.54** | 1.21 | 0.63 | **0.33** | 1.14 | **0.45** | 0.76 |
|  | 12 hours | 0.71 | 0.68 | 1.35 | **0.58** | 1.01 | 1.43 | **0.50** | 0.76 | **0.59** | 0.78 |
|  | 24 hours | **0.43** | **0.56** | 1.45 | 1.07 | 0.90 | **0.88** | **0.37** | 2.42 | **0.26** | **0.32** |
|  | 48 hours | **0.20** | **0.41** | 0.68 | **0.42** | 0.74 | **0.27** | **0.22** | **0.43** | **0.49** | **0.17** |
| ***MMP1*** | 6 hours | 0.73 | 0.83 | **0.64** | 0.77 | 1.08 | 0.73 | **0.30** | 1.01 | 0.91 | 0.76 |
|  | 12 hours | **0.50** | **0.33** | **0.45** | **0.67** | 0.82 | 3.94 | **0.58** | 0.67 | **0.69** | **0.56** |
|  | 24 hours | **0.18** | **0.30** | **0.49** | **0.16** | 0.63 | **0.61** | **0.17** | **0.41** | **0.24** | **0.14** |
|  | 48 hours | **0.11** | **0.16** | **0.23** | **0.17** | **0.45** | **0.31** | **0.20** | **0.24** | **0.42** | **0.10** |
| ***TIMP1*** | 6 hours | **5.55** | **2.21** | 1.28 | 1.39 | 1.21 | 1.01 | 0.97 | **2.11** | 1.08 | **1.42** |
|  | 12 hours | **7.85** | **2.15** | **1.61** | **1.45** | 1.34 | 0.57 | **1.79** | **2.15** | **1.57** | **1.85** |
|  | 24 hours | **6.73** | **3.34** | **1.66** | **1.85** | **1.52** | **3.23** | **1.85** | **4.69** | **2.04** | **2.49** |
|  | 48 hours | **11.30** | **3.40** | **2.31** | **1.75** | **2.00** | **3.92** | **3.95** | **2.57** | **1.83** | **1.38** |
| ***COL2A1*** | 6 hours | 1.21 | **4.56** | 1.02 | **1.60** | 1.47 | 0.91 | 1.10 | 1.31 | 1.31 | **2.05** |
|  | 12 hours | **1.69** | **2.91** | **1.79** | **1.71** | **2.61** | 0.92 | 0.94 | 2.48 | **1.42** | **3.81** |
|  | 24 hours | **2.70** | **3.95** | **2.80** | **2.09** | **4.01** | **9.97** | **2.27** | 1.04 | **2.65** | **8.26** |
|  | 48 hours | **5.67** | **1.72** | **3.84** | **2.91** | **2.70** | **10.24** | **2.00** | **1.16** | **5.38** | **5.65** |
| ***ACAN*** | 6 hours | 1.21 | 1.25 | 0.83 | 1.20 | 1.10 | 0.64 | 0.85 | 1.01 | 1.01 | 0.77 |
|  | 12 hours | 1.50 | 0.88 | 0.89 | 1.40 | 0.89 | 0.80 | **0.53** | 0.71 | 0.78 | 0.91 |
|  | 24 hours | **0.59** | 0.58 | **0.36** | 0.95 | **0.45** | **0.83** | **0.50** | 0.87 | **0.61** | 0.97 |
|  | 48 hours | **0.43** | **0.29** | **0.45** | **0.54** | **0.37** | **0.75** | **0.45** | **0.64** | **0.36** | **0.51** |
| ***SOX9*** | 6 hours | 1.13 | 1.18 | 0.87 | 0.80 | 0.94 | 1.01 | 0.58 | 0.95 | 0.95 | 0.79 |
|  | 12 hours | 1.38 | 0.86 | 0.67 | 0.79 | 0.65 | 1.18 | 0.81 | **0.81** | **0.73** | 0.91 |
|  | 24 hours | **0.25** | 0.86 | **0.45** | **0.59** | 0.76 | 0.91 | **0.72** | 0.63 | **0.81** | **0.74** |
|  | 48 hours | **0.36** | **0.52** | 0.55 | **0.55** | 0.77 | 1.31 | **0.81** | 0.69 | **0.89** | **0.76** |
