# Supplementary material for: Human Chondrocytes Respond Discordantly to the Protein Encoded by the Osteoarthritis Susceptibility Gene GDF5
Source: PLoS One. 2014 Jan 21;9(1):e86590. doi: 10.1371/journal.pone.0086590 (PMC3897745; doi:10.1371/journal.pone.0086590)
Supplement: Table S13 — The changes in expression of the target genes following OA chondrocyte micromass culturing and stimulation with TGF-β1. The chondrocytes from ten OA patients were cultured with or without TGF-β1 and gene expression was measured 5 days post stimulation. The actual values of any significant (P≤0.05, two-tailed Student’s t-test) fold changes in expression of the six target genes in response to the stimulation are shown in bold text. A value greater than 1 denotes an up regulation of gene expression and a value less than 1 denotes a down regulation of gene expression. (DOCX) [file pone.0086590.s017.docx]

**Table S13.** The changes in expression of the target genes following OA chondrocyte micromass culturing and stimulation with TGF-β1. The chondrocytes from ten OA patients were cultured with or without TGF-β1 and gene expression was measured 5 days post stimulation. The actual values of any significant (P≤0.05, two-tailed Student’s t-test) fold changes in expression of the six target genes in response to the stimulation are shown in bold text. A value greater than 1 denotes an up regulation of gene expression and a value less than 1 denotes a down regulation of gene expression.

| **Target gene** | **Patient** | | | | | | | | | |
| --- | --- | --- | --- | --- | --- | --- | --- | --- | --- | --- |
|  | **22** | **23** | **24** | **25** | **26** | **27** | **28** | **29** | **30** | **31** |
| ***MMP13*** | **0.71** | **0.30** | **0.26** | 0.48 | **0.25** | **0.30** | **0.83** | **0.85** | 1.00 | **0.71** |
| ***MMP1*** | **0.84** | **0.56** | **0.27** | **0.62** | **0.26** | **0.31** | **0.69** | 1.03 | **0.71** | **0.67** |
| ***TIMP1*** | **1.64** | 1.13 | **1.95** | **2.48** | **2.72** | **1.47** | 1.07 | 1.10 | **1.94** | **1.96** |
| ***COL2A1*** | 1.22 | **2.78** | **1.42** | **2.33** | 1.49 | **1.87** | **3.16** | **2.11** | **2.89** | **1.86** |
| ***ACAN*** | **1.71** | 1.18 | 1.13 | **2.87** | 1.10 | **1.43** | 1.09 | **1.35** | **1.17** | 1.08 |
| ***SOX9*** | **2.70** | **1.27** | 1.56 | 1.86 | **2.81** | **2.65** | 1.00 | **1.27** | **1.32** | **1.20** |
